# Marine Bioactive Peptides—Structure, Function, and Application 2.0

**DOI:** 10.3390/md23050192

**Published:** 2025-04-28

**Authors:** Bin Wang, Chang-Feng Chi

**Affiliations:** 1 Zhejiang Provincial Engineering Technology Research Center of Marine Biomedical Products, School of Food and Pharmacy, Zhejiang Ocean University, Zhoushan 316022, China; 2 National and Provincial Joint Engineering Research Centre for Marine Germplasm Resources Exploration and Utilization, School of Marine Science and Technology, Zhejiang Ocean University, Zhoushan 316022, China

In recent years, people’s lifestyles have undergone relatively significant changes. For instance, physical labor has decreased, and the number of people on a high-fat diet (HFD) has increased annually. This has also led to a year-on-year increase in the incidence and number of many diseases, such as non-alcoholic fatty liver disease (NAFLD), obesity, diabetes, hyperuricemia, etc. [1,2,3,4]. Therefore, people’s demands for food are gradually changing, shifting from merely solving the problem of basic sustenance to a rapid shift towards nutrition and functionality [5,6]. Therefore, the discovery and preparation of functional components from natural foods have become a research hotspot.

The ocean is rich in biological resources, and its unique ecological environment makes the active components in marine organisms significantly different from those in terrestrial organisms [7,8]. Therefore, scientists in the fields of life sciences, pharmacy, chemistry, and others around the world have focused their attention on marine bioactive substances, hoping to discover more functional molecules beneficial to human health from them. Among the numerous active components, marine bioactive peptides have also become the focus of research due to their high nutritional value and significant biological activity, such as anticancer, antioxidant, antimicrobial, anti-inflammatory, anti-photoaging, antidiabetic, antifreeze, and immune-modulating characteristics [9,10,11]. Therefore, we are launching the Special Issue “Marine Bioactive Peptides—Structure, Function, and Application 2.0” (https://www.mdpi.com/journal/marinedrugs/special_issues/5XPG3GP5G0) in May 2023, to highlight the latest research in the field of marine active peptides. Herein, we introduce a brief overview of the ten research papers and their findings contributed by the authors.

The development of high-value active peptide products by utilizing bulk marine biological resources and their processing by-products has always been the focus of research [12]. This can not only solve the environmental pollution caused by the by-products but also increase the profits of enterprises. Therefore, this Special Issue begins with the article by Wang et al. dedicated to the structural characteristics and bioactivity stability of protein hydrolysates (CFHs) from *Cucumaria frondosa* intestines and ovum [13]. In this study, the effects of alcalase, papain, flavourzyme, and neutrase on the structural characteristics and antioxidant stability of CFHs are systematically investigated, and the selected enzyme is vital to the physicochemical properties and biological activities of the bioactive hydrolysates produced. According to the degree of hydrolysis (DH), primary structures, surface hydrophobicity, antioxidant activity, pancreatic lipase inhibitory activity, and the stability of CFHs, the authors found that flavourzyme is the optimal choice for the production of CFHs applied in functional foods because the flavourzyme-prepared CFHs had the highest DH and exhibited the highest DPPH radical scavenging activity, reduction capacity, and pancreatic lipase inhibitory activity. Moreover, flavourzyme-prepared CFHs showed excellent antioxidant stability and pancreatic lipase inhibitory activity during simulated gastrointestinal digestion in vitro. These findings, especially the significant antioxidant and lipid-lowering activities of CFHs, strongly support their application in functional foods, dietary supplements, and nutritional health products. This research has developed a new application for the intestines and ovum of *C. frondosa*, which can improve the comprehensive utilization of marine biological resources.

The number of diabetes patients worldwide with diabetes is as high as 589 million (accounting for 11.1%), which is equivalent to approximately one in every nine people. It is estimated that by 2050, the total number of adult patients with diabetes worldwide will increase to 853 million (accounting for 13.0%) [14]. Therefore, the treatment of diabetes, especially the development of new diabetes drugs, has attracted much attention. The second article by Lin et al. concerns the antidiabetic effect of collagen peptides (HNCP, Mw < 1 kDa) from *Harpadon nehereus* bones [15]. The authors found that HNCP can significantly reduce blood glucose levels by increasing insulin secretion and glucose tolerance in streptozotocin-induced type 1 diabetic mice. Research on the mechanism of action has proved HNCP’s capability to improve antioxidant abilities by regulating the Keap1/Nrf2 pathway to increase the activity of antioxidant enzymes. Furthermore, HNCP can markedly ameliorate the glucose metabolism of type 1 diabetic mice by controlling the levels of glycosynthesis- and gluconeogenesis-related enzymes. This study explores the mechanism of HNCP in the treatment of diabetes from two aspects: oxidative stress and abnormal glucose metabolism, which are closely related to the occurrence and development of diabetes. This is an instructive study for the development of diabetes drugs.

Antarctic krill (*Euphausia superba*) has huge biomass and is regarded as the largest animal protein resource in the world [16]. Calcium (Ca) deficiency in the human body significantly influences cellular proliferation, neurotransmission, blood coagulation, muscle contraction, etc., and currently affects the health of about 900 million people in China [17]. The third article by Ge et al. concerns the preparation, characterization, and Ca absorption efficiency of Ca-chelating peptides from Antarctic krill protein hydrolysate [18]. In this study, the authors purify and identify 14 Ca-chelating peptides with the tetrapeptide (VERG), showing the highest chelating capability with Ca^2+^ in the groups of N-H, C=O, and -COOH. In addition, the VERG-Ca chelate remained stable in the simulated gastrointestinal digestion in vitro, and the monolayer experiment of Caco-2 cells proved that the VERG-Ca chelate could significantly improve calcium transport. The research results show that Ca-chelating peptides of Antarctic krill can be used as functional components to improve the bioavailability of Ca in healthy foods.

Metabolic syndrome (MetS) encompasses a series of metabolic abnormalities, such as hypertension, elevated triglycerides, hyperglycemia, low high-density lipoprotein cholesterol, and central obesity, and is associated with diabetes, cancer, neurodegenerative diseases, cardiovascular and cerebrovascular diseases, and NAFLD [19]. Focusing on improving diet to prevent MetS is a crucial aspect of enhancing human health. Therefore, the fifth article by Bjerknes et al. studies the glycemic regulatory characteristics of the protein hydrolysate (SPH) of Atlantic salmon (*Salmo salar*) through dipeptidyl peptidase-IV (DPP-IV) inhibition activity and the efficiency of direct glucose uptake in vitro [20]. The results indicate that SPH, especially the low-molecular-weight component prepared by membrane filtration (MW < 3 kDa), has a significant inhibitory effect on DPP-IV and can significantly increase the glucose uptake of L6 rat skeletal muscle cells, indicating that short-chained bioactive peptides in SPH mediate glucoregulatory activity. This study proves that the biotransformation of salmon processing by-products can provide high-quality nutrition and effective glycemic regulatory peptides, thereby significantly reducing the growing health burden of MetS. Moreover, bioactive peptides are promising functional tools that could be applied to future personalized medicine.

(-)-Doliculide is a marine cyclodepsipeptide isolated from *Dolabella auricularia* [21]. Due to its strong cytotoxicity, (-)-Doliculide has aroused the interest of researchers in the field of synthetic chemistry. Based on the structural characteristics of (−)-Doliculide, which is composed of peptide segments and polyketone segments, the fifth article by Tost and Kazmaier utilizes Matson’s homology method and focuses on post-modification at the end positions of (−)-Doliculide polyketone fragments, successfully synthesizing various polyketone derivatives of (−)-Doliculide. In addition, the author also studies the activity of the synthesized (−)-Doliculide polyketone derivatives. The results prove that all modifications of the i-Pr fragment led to the inactivity of (−)-Doliculide polyketone derivatives against HepG2 [22]. This study lays a methodological foundation for the subsequent research of (-)-Doliculide, including the structure–activity relationship of its derivatives.

Antimicrobial peptides (AMPs), regarded as host defense peptides (HDPs), are small in size with a MW less than 10 kDa and can be encoded in the genome or produced by hydrolyzing proteins or larger polypeptides. Moreover, AMPs exhibit a variety of biological activities, such as antimicrobial, antifungal, antioxidant, antihypertensive, immunomodulatory, and anticoagulant properties [23]. Therefore, the sixth article by Álvarez et al. concerns the identification, characterization, and bioactivity evaluation of AMPs from the mucus of *Seriola lalandi* and *Seriolella violacea* [24]. Nine peptides with a disordered or random coil secondary structure were prepared and identified using chromatography and mass spectrometry techniques. The analysis of the structure–activity relationship indicated that the antimicrobial activity of prepared peptides is closely associated with basic and aromatic amino acid residues, while cysteine residue could remarkably enhance the peptides’ antioxidant activity. In addition, the peptides with the highest antimicrobial activity also presented stimulated respiratory bursts in leukocytes. This study confirms the existence of bioactive peptides in the epidermal mucus of Chilean marine fish, providing a new source for the development of marine peptides.

Fatigue is a temporary decline in the functions of the body or brain due to continuous energy consumption, stress, or disease. It is divided into physiological fatigue and pathological fatigue [25]. Short-term fatigue can be relieved by rest, while long-term fatigue may be related to diseases and requires comprehensive intervention. High Fischer ratio oligopeptides (HFOs) are defined as peptide mixtures (2–9 peptides) with an F-ratio (branched-chain amino acids/aromatic amino acids) greater than 20. Due to their significant physiological activities, HFOs have aroused widespread interest and have been produced from grains, beans, meat, eggs, milk, and marine organisms [26]. Therefore, the seventh paper by Mao et al. systematically investigates the anti-fatigue function and mechanism of HFOs from Antarctic Krill (HFOs-AK) on exercise-induced fatigue [27]. HFOs-AK can significantly prolong the endurance of swimming time, improve physiological indicators, decrease metabolite levels, and protect the muscle tissues of exhausted mice. The research on the mechanism of action indicated that HFOs-AK could regulate AMPK and Keap1/Nrf2/ARE signaling pathways and ameliorate the energy metabolism and oxidative stress caused by intense exercise. In addition, HFOs-AK could improve the activity of Na^+^-K^+^-ATPase and Ca^2+^-Mg^2+^-ATPase to reduce tissue damage and increase ATP levels. Therefore, HFOs-AK can act as dietary ingredients applied in functional foods to resist the fatigue caused by intense exercise.

Melanin is a phenolic polymer that is widely present in animals and plants. Excessive melanin deposition can lead to a series of skin diseases, such as black and brown spots, and even cause melanoma and other skin cancers. Tyrosinase is the key enzyme for oxidizing tyrosine to generate melanin [28]. Therefore, screening safe and efficient tyrosinase inhibitors can effectively control melanin production. Therefore, the eighth article by Logesh et al. concerns the identification and mechanism of the tyrosinase inhibitory peptide AHYYD from *Pinctada martensii* nacre [29]. In this study, AHYYD, with an IC_50_ value of 2.012 ± 0.088 mM, showed significant tyrosinase inhibitory activity through a reversible competitive model. In addition, AHYYD had a strong binding affinity to tyrosinase with a binding energy of −8.0 kcal/mol. Moreover, AHYYD could significantly decrease the melanin content by inhibiting tyrosinase activity and positively increasing the activity of antioxidant enzymes in mouse B16F10 melanoma cells. These findings suggest that AHYYD has the potential to be applied to cosmetic products due to its positive therapeutic efficacy in decreasing intracellular melanin production.

The physical health of more than 1.13 billion people worldwide is affected by hypertension. The angiotensin-I converting enzyme (ACE) is a key enzyme in the renin–angiotensin–aldosterone regulatory system (RAAS), which catalyzes the conversion of inactive angiotensin I (Ang I) into potent Ang II, thereby inactivating the vasodilator bradykinin [30]. Therefore, screening for small molecule compounds to inhibit ACE activity has become an effective method for the treatment of hypertension. The ninth article by Li et al. concerns ACE inhibitory peptides from the protein hydrolysate of *Ulva prolifera* [31]. Then, an anti-ACE peptide KAF with an IC_50_ value of 0.63 ± 0.26 µM was separated and identified from the *U. prolifera* protein. KAF competes to bind ACE through two hydrogen bonds and inhibits its activity. Moreover, KAF could act on LTCC and RyR to increase Ca^2+^ levels in the endoplasmic reticulum of HUVECs and activate eNOS to promote the production of NO by regulating the Akt signaling pathway.

Saturated fatty acids play a significant role in maintaining human physiological functions. However, high concentrations of free fatty acids cause fat accumulation in the liver, promote the release of inflammatory factors and cellular dysfunction, induce liver cell damage and apoptosis, and thereby lead to the progression of NAFLD [32]. Therefore, the tenth paper by Song et al. investigates the effects of LALFVPR, KLHDEEVA, and PSRILYG from the bone collagen of *H. nehereus* on sodium palmitate (PANa)-induced hyperlipidemia in HepG2 cells [33]. The results demonstrate that LALFVPR, KLHDEEVA, and PSRILYG, particularly LALFVPR, can protect HepG2 cells against PANa-induced damage by upregulating the level of Nrf2 to enhance the antioxidant enzyme activity. Compared with LALFVPR and PSRILYG, LALFVPR showed better effects in reducing oxidative stress and lipid accumulation by regulating the expression levels of proteins closely related to lipid metabolism, such as FASN, ACC1, ATGL, and CPT1. In addition, the inhibitory ability of LALFVPR on pancreatic lipase activity is stronger than that of orlistat. This research provides support for the functional food of fish bone collagen peptides used in the treatment of hyperlipidemia.

The papers in this Special Issue contain studies on bioactive peptides from *C. frondosa*, *H. nehereus*, Antarctic krill (*E. superba*), Atlantic Salmon (*S. salar*), the mucus of *S. lalandi* and *S. violacea* to *P. martensii* and *U. prolifera*. In addition, the derivatives of (−)-Doliculide were synthesized. Moreover, it is worth noting that more than half of the included papers focus on metabolism-related diseases such as hypertension, hyperlipidemia, and diabetes. This indicates that metabolism-related diseases require more highly effective drugs, and the significant activity of marine peptides in this regard shows their potential application value.

In conclusion, the Guest Editors thank all the authors who contributed to this Special Issue, all the reviewers for evaluating the submitted manuscripts, and the Editorial Board of *Marine Drugs*, especially Jane Qiao, Assistant Editor of this journal, for their continuous help in making this Special Issue a reality.
